# Actin-dependence of the chloroplast cold positioning response in the liverwort *Marchantia polymorpha* L.

**DOI:** 10.7717/peerj.2513

**Published:** 2016-09-28

**Authors:** Shun Kimura, Yutaka Kodama

**Affiliations:** Center for Bioscience Research and Education, Utsunomiya University, Utsunomiya, Tochigi, Japan

**Keywords:** chloroplast movement, bryophyte, chloroplast cold positioning, actin, cytoskeleton, bioimaging, nucleus, peroxisome, organelle, plant cell

## Abstract

The subcellular positioning of chloroplasts can be changed by alterations in the environment such as light and temperature. For example, in leaf mesophyll cells, chloroplasts localize along anticlinal cell walls under high-intensity light, and along periclinal cell walls under low-intensity light. These types of positioning responses are involved in photosynthetic optimization. In light-mediated chloroplast positioning responses, chloroplasts move to the appropriate positions in an actin-dependent manner, although some exceptions also depend on microtubule. Even under low-intensity light, at low temperature (e.g., 5°C), chloroplasts localize along anticlinal cell walls; this phenomenon is termed chloroplast cold positioning. In this study, we analyzed whether chloroplast cold positioning is dependent on actin filaments and/or microtubules in the liverwort *Marchantia polymorpha* L. When liverwort cells were treated with drugs for the de-polymerization of actin filaments, chloroplast cold positioning was completely inhibited. In contrast, chloroplast cold positioning was not affected by treatment with a drug for the de-polymerization of microtubules. These observations indicate the actin-dependence of chloroplast cold positioning in *M. polymorpha*. Actin filaments during the chloroplast cold positioning response were visualized by using fluorescent probes based on fluorescent proteins in living liverwort cells, and thus, their behavior during the chloroplast cold positioning response was documented.

## Introduction

Chloroplasts change their subcellular localization in response to alterations in the environment such as ambient light and temperature (Senn, 1908). In the light-mediated response of leaf mesophyll cells, under high-intensity (strong) light conditions, chloroplasts localize along anticlinal cell walls due to avoidance from the strong light (avoidance response). In contrast, under low-intensity (weak) light conditions, chloroplasts localize along periclinal cell walls due to accumulation to the weak light (accumulation response). Dark-induced positioning is also known; for instance, chloroplasts localize to the bottom of cells in flowering plants such as *Arabidopsis thaliana* (Suetsugu, Kagawa & Wada, 2005), and along the anticlinal cell walls in the liverwort *Marchantia polymorpha* (Komatsu et al., 2014), and the fern *Adiantum capillus-veneris* (Kagawa & Wada, 1996). Chloroplast positions also change with temperature alterations. When the temperature was shifted from room temperature (e.g., 20°C) to low temperature (e.g., 5°C), chloroplasts re-localize from periclinal cell walls to anticlinal cell walls, even if under weak light conditions. Over one century ago, cold-induced chloroplast relocation movements (cold positioning) were analyzed in the moss *Funaria hygrometrica* (Senn, 1908). Recently, we also found that cold positioning occurs in the ferns *A. capillus-veneris* and *Pteris cretica* as well as the liverwort *M. polymorpha* (Kodama et al., 2008; Ogasawara et al., 2013). Although a blue-light photoreceptor, phototropin2, was reported to mediate chloroplast cold positioning in *A. capillus-veneris* (Kodama et al., 2008), other factors involved in cold positioning have not been found.

It is well known that actin cytoskeletal filaments are involved in light-induced chloroplast positioning (Gabryś, 2004; Wada & Suetsugu, 2004; Suetsugu & Wada, 2007; Kong & Wada, 2011). The anti-actin drugs (actin polymerization inhibitors) such as cytochalasin and latrunculin, which depolymerize actin filaments, prevent light-induced chloroplast positioning in green alga and vascular plants (Wagner, Haupt & Laux, 1972; Malec, Rinaldi & Gabryś, 1996; Kandasamy & Meagher, 1999). Recently, in a study of light-induced chloroplast positioning, short actin filaments were found on the chloroplast periphery in transgenic *A. thaliana* expressing a genetically encoded fluorescent probe (GFP-mTalin) that binds to actin filaments (Kadota et al., 2009). The short actin filaments are now called chloroplast actin (cp-actin) filaments. Cp-actin filaments evenly distribute at the periphery of unmoving chloroplasts; they reorganize on the chloroplast in response to light before and during relocation with a biased distribution of cp-actin filaments at the front region of the chloroplast (Kadota et al., 2009). Cp-actin filaments exhibit rapid dynamic changes during light-induced chloroplast positioning (Kadota et al., 2009; Kong et al., 2013), therefore, they seem to be an important machinery in the light-induced chloroplast positioning observed in *A. thaliana* (Kadota et al., 2009), *A. capillus-veneris* (Tsuboi & Wada, 2012) and the moss *Physcomitrella patens* (Yamashita et al., 2011). In *P. patens*, an involvement of microtubules in addition to actin filaments has been reported in light-induced chloroplast positioning (Sato, Wada & Kadota, 2001). When *P. patens* is exposed to the anti-microtubule drug (microtubule polymerization inhibitor) Cremart, the red-light-induced chloroplast positioning response was completely inhibited (Sato, Wada & Kadota, 2001). Blue-light-induced chloroplast positioning in *P. patens* was partially inhibited by Cremart, and completely inhibited by simultaneous treatment of Cremart and cytochalasin B (Sato, Wada & Kadota, 2001). However, the molecular mechanism of temperature-dependent chloroplast positioning is not completely understood, and it is unknown whether the chloroplast cold positioning response is mediated via cytoskeletal filaments such as actin and/or microtubules.

In this study, we used de-polymerization drugs and cytoskeletal filament fluorescent probes to determine whether chloroplast cold positioning is dependent on actin filaments and/or microtubules in the liverwort *M. polymorpha*.

## Materials and Methods

### Plant materials and growth conditions

The male strain (Tak-1) of *M. polymorpha* was used in this study. As previously described (Ogasawara et al., 2013), the thalli were cultured on M51C medium with 1% agar (M51C agar), and asexually maintained under 75 μmol photons *m*^−2^*s*^−1^ continuous white light (FL40SW, NEC Corporation, Tokyo, Japan). One-day-old gemmalings (immature thalli grown from gemmae) obtained from approximately 1-month-old wild type or transgenic thalli (G1 generation) were used for all experiments.

### Treatments of temperature, light, and inhibitors

One-day-old gemmalings were incubated at 22°C or 5°C in the temperature-controlled incubators (IJ100 or IJ101, Yamato Scientific Co., Ltd., Tokyo, Japan). In the incubators, illuminators with white- or blue-colored light-emitting diodes (LEDs) (OptoSupply Limited, Hong Kong, China) were set-up to irradiate the gemmalings with weak light (Ogasawara et al., 2013; Y Fujii et al., 2016, unpublished data). The gemmalings were incubated at 22°C for 2 h followed by transfer at 5°C for 24 h to induce chloroplast cold positioning.

Before induction of chloroplast cold positioning, an inhibitor (1-μM latrunculin A, 50-μM cytochalasin B, or 10-μM oryzalin) was applied at 22°C for 2 h. To prepare the drugs with appropriate concentrations for use, stock solutions stored at −20°C were diluted with sterile water. The stock solutions were 0.1-mM latrunculin A in ethanol, 20-mM cytochalasin B in dimethyl sulfoxide (DMSO) protected from light, and 10-mM oryzalin in DMSO protected from light.

For analysis of chloroplast accumulation response, dark positioning response of chloroplast was induced under dark at 22°C for 4 days, and then the cells were treated with an inhibitor, 1-μM latrunculin A or 10-μM oryzalin, under the dark at 22°C for 2 h. The treated cells were transferred to weak blue-light (25 μmol photons *m*^−2^*s*^−1^) condition and incubated for 12 h to induce chloroplast accumulation response. For analysis of chloroplast avoidance response, chloroplast accumulation response was induced under weak blue-light (25 μmol photons *m*^−2^*s*^−1^) condition at 22°C for 24 h, and the cells were treated with an inhibitor, 1-μM latrunculin A or 10-μM oryzalin, under the weak light condition at 22°C for 2 h. The treated cells were transferred to strong blue-light (50 μmol photons *m*^−2^*s*^−1^) condition and incubated for 3 h to induce chloroplast avoidance response.

### Plasmid constructions

To visualize actin filaments and microtubules in *M. polymorpha*, binary vectors adapted to the gateway cloning system (Invitrogen, CA, USA) (Ishizaki et al., 2015a.) were used to perform *Agrobacterium*-mediated transformations.

For Lifeact-Citrine, the cDNA fragment for the Lifeact peptide (MGVADLIKKFESISKEE) (Riedl et al., 2008) was produced by PCR with the following oligo primers: [5′-GGGGACAAGTTTGTACAAAAAAGCAGGCTTCATGGGCGTGGCCGACCTGATC AAGAAGTTCGAGAGCATC-3′ and 5′-ACCTCCAGAGCCACCCTCCTCCTTGCTGATG CTCTCGAACTT-3′]. The cDNA fragment for Citrine was amplified by PCR with pDONR207-Citrine (Tsuboyama & Kodama, 2014) as a template with the following oligo primers: [5′-GGTGGCTCTGGAGGTATGGTGAGCAAGGGC-3′ and 5′-GGGGACCACTTTGTACAAGAAAGCTGGGTCTCACTTGTACAGCTCGTCC-3′]. The two fragments for Lifeact and Citrine were mixed, and this solution was then used as a template for the second PCR to fuse the two fragments with the following oligo primers: [5′-GGGGACAAGTTTGTACAAAAAAGCAGGCTTCATGGGCGTGGCCGAC CTGATCAAGAAGTTCGAGAGCATC-3′ and 5′-GGGGACCACTTTGTACAAGAAAG CTGGGTCTCACTTGTACAGCTCGTCC-3′]. The resulting fusion fragment for Lifeact-Citrine was cloned into the pDONR207 plasmid using the BP reaction, and the sequence was checked. There is no linker sequence between Lifeact and Citrine. The fusion gene for Lifeact-Citrine was transferred into pMpGWB403 (Ishizaki et al., 2015a.).

To construct the fusion gene for Citrine-mTalin, the cDNA fragment for mTalin was amplified by PCR with a template DNA containing the cDNA of mTalin with the following oligo primers: [5′-GGGGACAAGTTTGTACAAAAAAGCAGGCTTCAGCGGA GCAGGAGCAGGA-3′ and 5′-GGGGACCACTTTGTACAAGAAAGCTGGGTCTTAGTG CTCGTCTCGAAGC-3′]. The amplified fragment was cloned into the pDONR207 plasmid using the BP reaction, and the sequence was confirmed. The cloned gene for mTalin was transferred into the pMpGWB105 vector (Ishizaki et al., 2015a.), which harbors the *Citrine* gene,generating a *Citrine-mTalin* fusion gene. The amino acid sequence of a linker between Citrine and mTalin is (AVITSLYKKAGF).

For visualization of microtubules, the cDNA fragment for MpTubulin beta 3 (accession number: KJ948117) (Buschmann et al., 2016) was amplified by PCR with the cDNA library as a template using the following oligo primers: [5′-GGGGACAAGTTTGTACAA AAAAGCAGGCTTCATGAGAGAAATTCTCCAC-3′ and 5′-GGGGACCACTTTGTACAA GAAAGCTGGGTCTTAGTTGGCTTCAAGCTCT-3′]. The cDNA library prepared from the *M. polymorpha* Tak-1 strain was used. The amplified cDNA fragment for MpTubulin was cloned into the pDONR207 plasmid using the BP reaction. After the sequence was checked, the fragment was transferred into the pMpGWB105 vector (Ishizaki et al., 2015a.), which harbors the *Citrine* gene, generating a *Citrine-MpTubulin* fusion gene. The amino acid sequence of a linker between Citrine and MpTubulin is (AVITSLYKKAGF).

### Genetic transformation of *Marchantia polymorpha*

*Agrobacterium*-mediated genetic transformation of *M. polymorpha* was performed by G- and T-AgarTrap procedures (Tsuboyama-Tanaka & Kodama, 2015; Tsuboyama-Tanaka, Nonaka & Kodama, 2015). In this study, Tak-1 was used as the material to produce these transformants. For all experiments, transgenic G2 gemmae were used. Several transformants were independently produced for each construct (*Lifeact-Citrine*, *Citrine-mTalin* or *Citrine-MpTubulin*), and it was confirmed that the chloroplast cold positioning response was normally induced in these transformants.

### Microscopic observation and analysis

Observation of chloroplast position in gemmaling under a stereo fluorescence microscope using an MZ16F system (Leica Microsystems, Wetzlar, Germany) has been reported previously (Ogasawara et al., 2013). Excitation filter 480/40 nm and barrier filter LP 510 nm were used for observation, and the P/A ratio method (Kodama et al., 2008; Ogasawara et al., 2013) was utilized for evaluation of chloroplast positioning in *M. polymorpha*. Because procedure of the P/A ratio method for *M. polymorpha* was previously reported (Ogasawara et al., 2013), we briefly describe in this paper. Chloroplast position was quantified by the brightness ratio of chlorophyll fluorescence from chloroplasts along the anticlinal and periclinal cell walls. Fluorescent intensities from 30 points (0.625 mm each) and 30 areas (39.1 mm^2^ each) were measured along the anticlinal and periclinal cell walls, respectively. After subtraction of background fluorescence from each of the averaged fluorescent intensities, the P/A ratio with a standard deviation was obtained as an average of experiments repeated five times (the raw data in Data S1).

To visualize the fluorescence of Lifeact-Citrine and Citrine-mTalin for actin filaments and Citrine-MpTubulin for microtubules, a confocal laser scanning microscope SP8X system (Leica Microsystems, Wetzlar, Germany) was used with a 514-nm laser obtained from a highly flexible pulsed white-light laser. Citrine and chlorophyll fluorescence were detected by the hybrid detector and the conventional photomultiplier tube, respectively (Kodama, 2016). To reject chlorophyll autofluorescence when Citrine fluorescence was observed, the time-gated imaging method (Kodama, 2016) was employed with a gating time set at 0.5–12.0 ns. For capturing images, the scan speed was set at 100 Hz (100 lines/s) and line averages were four times. To visualize the fluorescence of Citrine in the nucleus and peroxisomes (Ogasawara et al., 2013), another confocal laser scanning microscope SP2 system (Leica Microsystems, Wetzlar, Germany) was used with a 514-nm laser (Argon laser).

## Results and Discussion

### Effects of inhibitors for cytoskeletal filaments in *M. polymorpha*

To determine whether chloroplast cold positioning is dependent on actin filaments and/or microtubules, we treated gemmalings (thallus grown from gemma) with anti-actin and anti-microtubule drugs, and observed the chloroplast cold positioning response in the cells. The degree of the response was evaluated by the P/A ratio method, which is a quantitative method for chloroplast positioning (Kodama et al., 2008; Ogasawara et al., 2013). Chloroplast position was quantified by the brightness ratio of chlorophyll fluorescence from chloroplasts along the anticlinal and periclinal cell walls, and chloroplast position was stated as the numeric value, P/A ratio. A procedure of the P/A ratio method for *M. polymorpha* was previously reported (Ogasawara et al., 2013).

**Figure 1:**
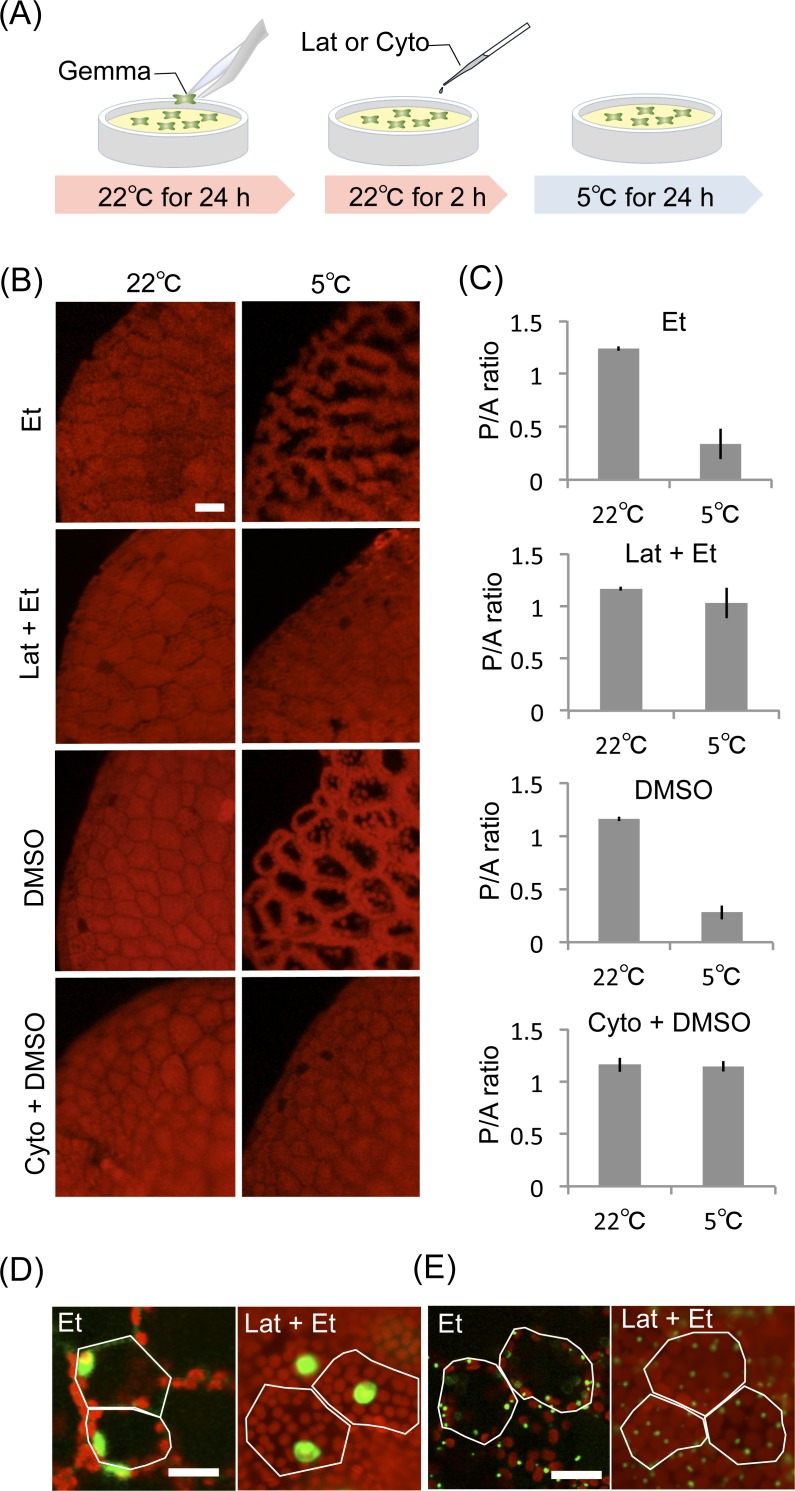
Effect of inhibitors for actin filaments in organelle cold positioning. (A) Procedure of inhibitor treatments for actin filaments. (B) Representative images of chloroplast position with the inhibitors, 1-μM latrunculin A (Lat) and 50-μM cytochalasin B (Cyto). As controls, 1% ethanol (Et) and 0.25% DMSO were used. Chloroplast position was visualized with chlorophyll fluorescence in red. Scale bar represents 50 μm. (C) Quantification of chloroplast position (B) by measurement of the P/A ratio. Bars indicate standard deviations. (D) Representative images of cold-mediated intracellular positions of nuclei at 5°C, with 1-μM latrunculin A (Lat) as the inhibitor and 1% ethanol (Et) as a control. Scale bar represents 25 μm. Lines are drawn along the cell shape. Fluorescence of Citrine-labeled nuclei (Ogasawara et al., 2013) and chlorophyll were colored in green and red, respectively. (E) Representative images of cold-mediated intracellular positions of peroxisomes at 5°C. Scale bar represents 25 μm. Lines are drawn along the cell shape. Fluorescence of Citrine-labeled peroxisomes (Ogasawara et al., 2013) and chlorophyll were colored in green and red, respectively.

To analyze the involvement of actin filaments in chloroplast cold positioning, 1-day-old gemmalings cultured at 22°C for 24 h were pre-treated with 1-μM latrunculin A, an anti-actin drug, at 22°C for 2 h followed by incubation at 5°C for 24 h to induce cold positioning (Fig. 1A). The disruption of actin filaments with 1-μM latrunculin A was evaluated (see below). In the control experiment with 1% ethanol, cold positioning was typically induced (Fig. 1B), and the average P/A ratio changed from 1.2 at 22°C to 0.3 at 5°C (Fig. 1C). Conversely, in the gemmalings treated with 1-μM latrunculin A, cold positioning was not induced (Fig. 1B), and the average P/A ratio remained unchanged at 22°C and 5°C (Fig. 1C). To avoid unforeseen side effects of latrunculin A, another anti-actin drug (cytochalasin B) was also tested. Disruption of the actin filaments with 50-μM cytochalasin B was also confirmed in *M. polymorpha* (see below). When 1-day-old gemmalings were pre-treated with 50-μM cytochalasin B at 22°C for 2 h (Fig. 1A), cold positioning was completely inhibited (Figs. 1B and 1C). In the control experiment with 0.25% dimethyl sulfoxide (DMSO), we successfully observed the induction of cold positioning (Figs. 1B and 1C). These results indicate that the induction of the chloroplast cold positioning response is dependent on actin filaments in *M. polymorpha*. Our previous study reported that, in addition to chloroplasts, the nucleus and peroxisomes also change their subcellular localization in response to cold temperatures (Kodama et al., 2008; Ogasawara et al., 2013). Transformants of *M. polymorpha*, wherein the nuclei or peroxisomes are visualized by fluorescent proteins, have been previously produced (Ogasawara et al., 2013). In this study, the transformants were treated with 1-μM of the anti-actin drug latrunculin A. Similar to chloroplasts, latrunculin A inhibited cold-induced relocations of the nucleus and peroxisomes in *M. polymorpha* (Figs. 1D and 1E); thus, cold-induced organelle relocation appears to be mediated via actin filaments in *M. polymorpha*. Because light-induced positioning responses of nuclei and peroxisomes are dependent on light-induced positioning responses of the attached chloroplasts (Higa et al., 2014; Oikawa et al., 2015), the cold-induced relocations of the nuclei and peroxisomes might be dependent on the relocation of the chloroplasts in *M. polymorpha*.

**Figure 2:**
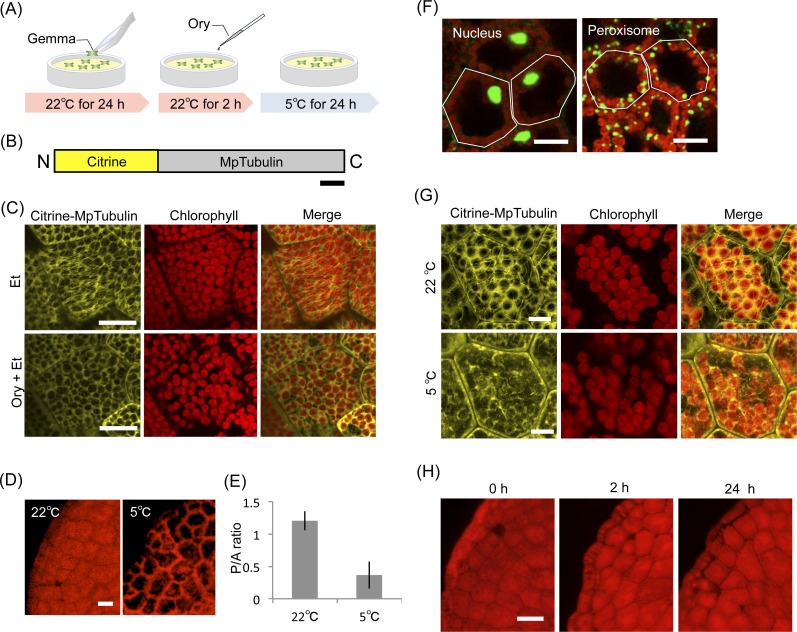
Effect of inhibitors for microtubules in organelle cold positioning. (A) Procedure of inhibitor treatments for microtubules. (B) Schematic illustration of Citrine-MpTubulin. N and C indicate the amino and carboxyl termini, respectively. Bar represents 50 amino acids. (C) Disruption of microtubules visualized by Citrine-MpTubulin with 10-μM oryzalin (Ory) treatment. Fluorescence of Citrine-MpTubulin and chlorophyll were colored in yellow and red, respectively. Scale bars represent 25 μm. (D) Representative images of chloroplast position with the inhibitor, 10-μM oryzalin (Ory). Scale bar represents 50 μm. (E) Quantification of chloroplast position (D) by measurement of the P/A ratio. Bars indicate standard deviations. (F) Representative images of cold-mediated intracellular positions of nuclei and peroxisomes at 5°C, with 10-μM oryzalin (Ory) as the inhibitor. Scale bars represent 25 μm. Lines are drawn along the cell shape. Fluorescence of Citrine-labeled organelles (nuclei and peroxisomes) (Ogasawara et al., 2013) and chlorophyll were colored in green and red, respectively. (G) Disruption of microtubules visualized by Citrine-MpTubulin at 5°C for 2 h. Scale bars represent 10 μm. (H) Chloroplast positioning after treatment of 10-μM oryzalin (Ory) at 22°C for 0 h, 2 h, or 24 h. Scale bar represents 50 μm.

**Figure 3:**
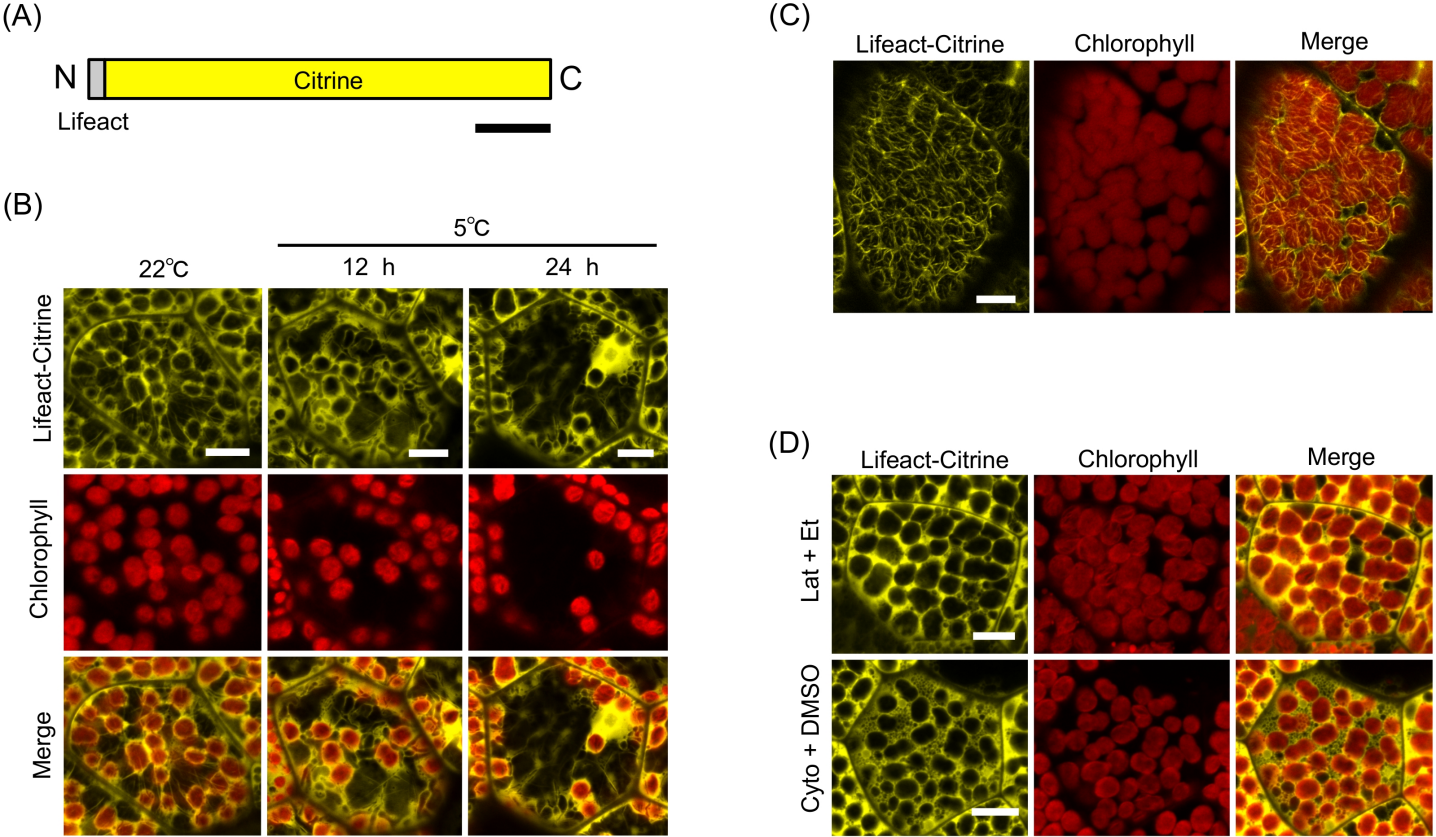
Lifeact-based visualization of actin filaments during chloroplast cold positioning. (A) Schematic illustration of Lifeact-Citrine. N and C indicate the amino and carboxyl termini, respectively. Bar represents 50 amino acids. (B) Visualization of actin filaments mediated by Lifeact-Citrine probe during chloroplast cold positioning in 1-day-old gemmaling. Fluorescence of Lifeact-Citrine and chlorophyll were colored in yellow and red, respectively. Scale bars represent 10 μm. (C) Visualization of actin filaments by Lifeact-Citrine probe in 4-day-old gemmaling. Scale bar represents 10 μm. (D) Disruption of actin filaments visualized by Lifeact-Citrine probe with treatment of 1-μM latrunculin A (Lat) and 50-μM cytochalasin B (Cyto). Scale bars represent 10 μm.

We next examined the effect of the anti-microtubule drug oryzalin on cold positioning (Fig. 2A). When transgenic gemmalings expressing Citrine-MpTubulin (Fig. 2B) were pre-treated with 10-μM oryzalin, disruption of microtubules was observed (Fig. 2C). MpTubulin-fused fluorescent protein has been previously reported to visualize microtubules in *M. polymorpha* (Buschmann et al., 2016). Note that the previous study used *β*-tubulin 1 from *M. polymorpha*, while we employed *β*-tubulin 3 from *M. polymorpha* (Buschmann et al., 2016). Treatments with oryzalin did not affect the cold positioning responses of chloroplasts (Figs. 2D and 2E), the nucleus or peroxisomes (Fig. 2F), indicating no involvement of microtubules in the cold positioning response.

Because microtubule is known to be cold sensitive (Hardham & Gunning, 1978), we investigated the behavior of microtubule in *M. polymorpha*. In transgenic gemmalings expressing Citrine-MpTubulin, cold-induced disruption of microtubules was observed after cold treatment for 2 h (Fig. 2G), suggesting no formation of microtubules during chloroplast cold positioning. In other words, disruption of microtubules may trigger to induce the relocation of chloroplast from periclinal to anticlinal cell walls. However, oryzalin-induced disruption did not change chloroplast positioning, although we observed for 24 h at 22°C (Fig. 2H). Taken together, we concluded that microtubule is not involved in the chloroplast cold positioning response in *M. polymorpha*.

### Visualization of actin filaments during chloroplast cold positioning

Based on the above pharmacological experiments, the induction of the chloroplast cold positioning response is dependent on actin filaments, but not microtubules, in *M. polymorpha*. During the light-induced chloroplast positioning in *A. thaliana, A. capillus-veneris*, and *P. patens*, the short cp-actin filaments that exhibit rapid dynamic changes on the chloroplast envelope were reported (Kadota et al., 2009; Yamashita et al., 2011; Tsuboi & Wada, 2012; Kong et al., 2013). To understand the behavior of actin filaments during cold positioning, actin filaments were visualized by using genetically encoded fluorescent probes in *M. polymorpha*. The Lifeact peptide, which is a short peptide of 17 amino acids known to bind to filamentous actin (F-actin) (Riedl et al., 2008), was previously reported to visualize actin filaments by fusing with a fluorescent protein in *M. polymorpha* (Era et al., 2009). In this study, we fused the Lifeact peptide with the yellow fluorescent protein Citrine (Lifeact-Citrine) (Fig. 3A) and transformed the fusion gene for Lifeact-Citrine into *M. polymorpha*. When 1-day-old transgenic gemmalings expressing the Lifeact-Citrine were observed at 22°C, many chloroplasts localized along periclinal cell walls, inducing the accumulation response (Fig. 3B). A network of long F-actin structures was slightly observed in the 1-day-old cells (Fig. 3B), and clearly observed in the 4-day-old cells (Fig. 3C). The structures were disrupted by anti-actin drugs (1-μM latrunculin A and 50-μM cytochalasin B), verifying the visualization of F-actin (Fig. 3D). However, around the chloroplasts in *M. polymorpha*, a liquid-like signal rather than a filamentous signal of Lifeact-Citrine was observed, and a cp-actin-like structure was not detected (Figs. 3B and 3C). When cold positioning was induced, the chloroplasts relocated from periclinal to anticlinal cell walls and the long F-actin structures at periclinal cell walls were reduced together with the chloroplasts (Fig. 3B). Eventually, we could not observe cp-actin-like structures around the chloroplast during the cold positioning response (Fig. 3B).

**Figure 4:**
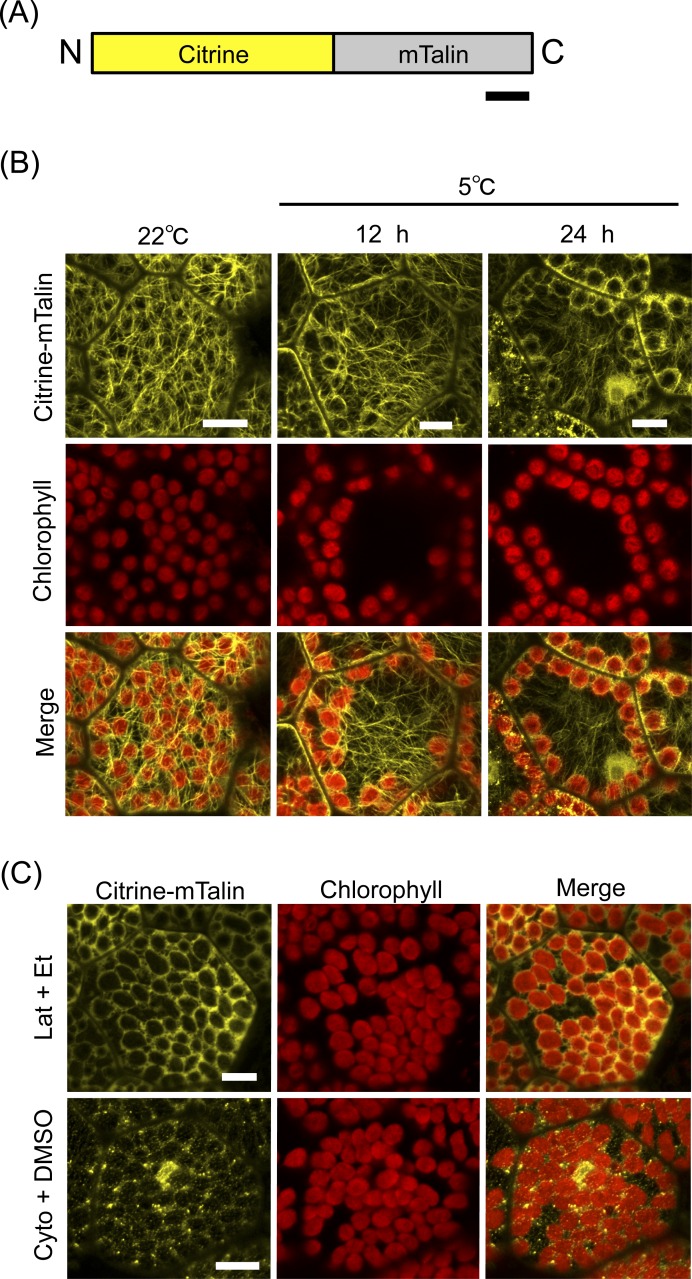
mTalin-based visualization of actin filaments during chloroplast cold positioning. (A) Schematic illustration of Citrine-mTalin. N and C indicate the amino and carboxyl termini, respectively. Bar represents 50 amino acids. (B) Representative images of actin filaments visualized by Citrine-mTalin during chloroplast cold positioning. Fluorescence of Citrine-mTalin and chlorophyll were colored in yellow and red, respectively. Scale bars represent 10 μm. (C) Disruption of actin filaments visualized by Citrine-mTalin with treatment of 1-μM latrunculin A (Lat) and 50-μM cytochalasin B (Cyto). Scale bars represent 10 μm.

A previous study of *A. thaliana* demonstrated that mouse talin (mTalin) (Kost, Spielhofer & Chua, 1998), which is another actin binding protein, is suitable for the visualization of cp-actin as compared with the Lifeact peptide (Kong et al., 2013). In the moss *P. patens*, cp-actin filaments have been successfully visualized by the mTalin-fused fluorescent protein (Yamashita et al., 2011). Therefore, mTalin was considered to be suitable for observing cp-actin filaments. We fused mTalin with Citrine (Citrine-mTalin) (Fig. 4A) and transformed Citrine-mTalin into *M. polymorpha* (Fig. 4B). Surprisingly, the actin filaments visualized by Citrine-mTalin were totally different from that by Lifeact-Citrine in the 1-day-old cells (Figs. 3B and 4B), and numerous long F-actin structures were clearly observed in the transgenic cells expressing Citrine-mTalin (Fig. 4B). In this context, a type of F-actin bound by Citrine-mTalin may differ from that bound by Lifeact-Citrine. In addition, the structure was disrupted by anti-actin drugs (1-μM latrunculin A and 50-μM cytochalasin B), visualizing F-actin with Citrine-mTalin (Fig. 4C). In the 1-day-old transgenic gemmalings expressing the Citrine-mTalin, the chloroplast accumulation response was induced at 22°C, and the long F-actin structures were visualized as likely covering the chloroplasts (Fig. 4B). However, as with the case of Lifeact-Citrine (Fig. 3B), cp-actin-like short F-actin structures were not detected by Citrine-mTalin (Fig. 4B). When cold positioning was induced, any biased distribution of the actin filaments between the front and rear of chloroplasts in its directional movement was also undetectable (Fig. 4B). However, the reduction of the long F-actin structures at periclinal cell walls was clearly observed during chloroplast relocation in the transgenic liverworts expressing Citrine-mTalin, confirming the results with Lifeact-Citrine (Fig. 4B).

In the present study, by using Lifeact-Citrine and Citrine-mTalin as fluorescent probes, we detected long F-actin structures, but not short F-actin structures, such as cp-actin filaments, around chloroplast in *M. polymorpha*. It remains to be known why these particular structures of cp-actin were not detected in *M. polymorpha*. It is possible that the actin-based machinery involved in the chloroplast positioning observed in *M. polymorpha* may differ from the already reported cp-actin filaments as cp-actin-independent but actin-mediated mechanisms for light-induced positioning have recently been suggested (Suetsugu et al., 2016). As a possibility, cp-actin filaments may not form in chloroplast cold positioning. Based on our observations (Fig. 3B and 4B), long F-actin structures at periclinal cell walls may be involved in chloroplast cold positioning of *M. polymorpha* but further experiments are necessary to make this conclusion.

**Figure 5:**
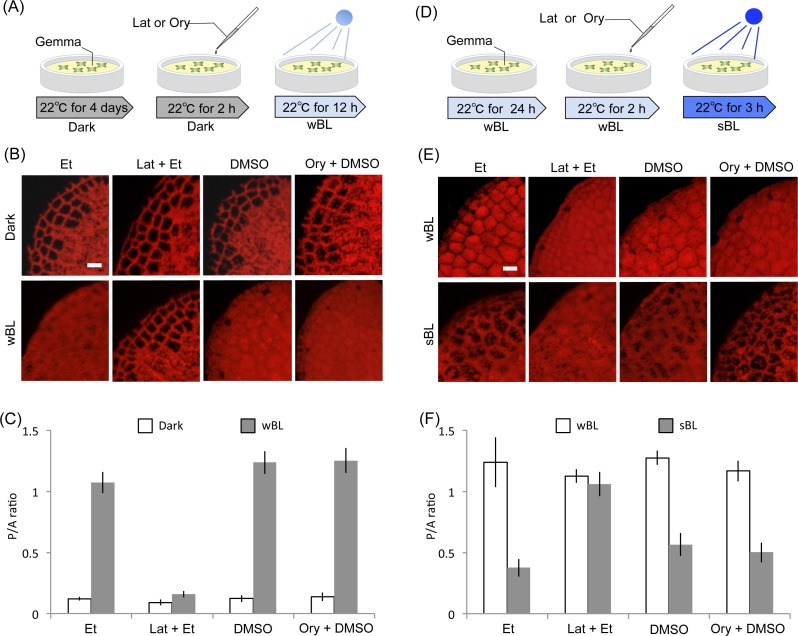
Actin-dependency in the light-induced chloroplast positioning responses. (A) Procedure of inhibitor treatments to analyze chloroplast accumulation response. wBL, weak blue-light. (B) Representative images of chloroplast position after induction of the accumulation response with the inhibitor, 1-μM latrunculin A (Lat) or 10-μM oryzalin (Ory). As controls, 1% ethanol (Et) and 0.1 % DMSO were used. Scale bar represents 50 μm. (C) Quantification of chloroplast position (B) by measurement of the P/A ratio. Bars indicate standard deviations. (D) Procedure of inhibitor treatments to analyze chloroplast avoidance response. wBL, weak blue-light; sBL, strong blue-light. (E) Representative images of chloroplast position after induction of the avoidance response with the inhibitor, Lat or Ory. As controls, 1% ethanol (Et) and 0.1% DMSO were used. Scale bar represents 50 μm. (F) Quantification of chloroplast position (E) by measurement of the P/A ratio. Bars indicate standard deviations.

Cp-actin filaments for chloroplast positioning have been found not only in the vascular plants, *A. thaliana* (the flowering plant) and *A. capillus-veneris* (the fern), but also in a bryophyte *P. patens* (the moss), and factors regulating cp-actin filaments are conserved in streptophytes (Suetsugu & Wada, 2016). Thus, the cp-actin filaments are believed to be conserved in many plants including *M. polymorpha*. Actually, the light-induced chloroplast positioning responses were dependent on actin filaments, but not microtubules, in *M. polymorpha* (Fig. 5). In our microscopic observation and P/A ratio evaluation, both light-induced chloroplast avoidance and accumulation responses were inhibited by latrunculin A, but not oryzalin (Fig. 5). Even if *M. polymorpha* has chloroplast positioning responses via regulation by cp-actin filaments, the structure would be different from the already reported cp-actin structure because there were no observations of analogous structure at both 22°C and 5°C in the present study (Figs. 3B and 4B). In order to determine this in *M. polymorpha*, particularly during temperature-induced chloroplast positioning responses, technical limitations of microscopic observation need to be overcome. In the previous study of *A. thaliana*, to analyze the light-induced response, cp-actin was observed by taking photographs (i.e., snapshot), and also by recording movies (i.e., time-lapse) (Kadota et al., 2009). In the recoded movie of the light-induced response, both imaging and microbeam irradiation were simultaneously performed (Kadota et al., 2009). In the present study, to analyze temperature-induced responses, we took only photographs because technically it was not possible to record movies with fluorescence images under cold conditions. In our experiments, cells were observed by confocal laser scanning microscopy after temperature treatment in a conventional incubator, suggestive of a time-lag (within minutes) between the treatment and the observation. The reorganization of cp-actin filaments has been reported to occur on a minute time-scale (Kadota et al., 2009), thus cp-actin filaments may have already disappeared in the cells when they were scanned in our study. To find the expected cp-actin filaments under temperature alteration, the use of temperature-controlled fluorescence microscopy or confocal microscope equipped with a time-lapse video recording system may be necessary. Fluorescence observation by confocal laser scanning microscopy under temperature controlled conditions has been reported previously (Holzinger et al., 2007), thus these technical limitations should be resolved in the future by adding a time-lapse recording system.

In summary, our findings indicate that chloroplast cold positioning is actin-dependent; however, the actin-based machinery (i.e., cp-actin-like machinery associated with chloroplasts or long F-actin structures along periclinal cell walls) necessary for the cold positioning response of *M. polymorpha* could not be conclusively identified in the present study. Future work will include temperature-controlled fluorescence microscopy with a time-lapse video recording system to determine the machinery. Currently, *M. polymorpha* is being developed as a model liverwort, and various molecular biological techniques such as transformation and genome editing have been developed (Ishizaki et al., 2015b). We believe that future research using these molecular techniques, in addition to temperature-controlled fluorescence microscopy, will allow the identification of the actin-based machinery critical for the cold positioning response.

##  Supplemental Information

10.7717/peerj.2513/supp-1 Data S1 Raw data for P/A ratio

## Additional Information and Declarations

### Data Availability

The following information was supplied regarding data availability: The raw data has been supplied as a Data S1.
